# Impact of a Formative Program on Transgender Healthcare for Nursing Students and Health Professionals. Quasi-Experimental Intervention Study

**DOI:** 10.3390/ijerph16173205

**Published:** 2019-09-02

**Authors:** Jesús Manuel García-Acosta, Maria Elisa Castro-Peraza, Ángeles Arias Rodriguez, María Luisa Perez-Cánovas, Maria Inmaculada Sosa-Alvarez, Rosa Llabrés-Solé, Ana María Perdomo-Hernández, Nieves Doria Lorenzo-Rocha

**Affiliations:** 1 Faculty of Nursing N. S. Candelaria, Canary Islands Public Health Service, University of La Laguna, 38200 Tenerife, Canary Islands, Spain; 2 Faculty of Health Sciences, University of La Laguna, 38200 Tenerife, Canary Islands, Spain

**Keywords:** motion pictures, problem-based learning, education, nursing, learning, transgender persons, gender identity

## Abstract

Background: The field of specific healthcare for transgender people has not been included in the official curriculum of professionals. This causes a lack of knowledge that can be presumed to become a barrier to healthcare. Currently, different methodologies are emerging to achieve meaningful learning for students and professionals. The objective of this study was to evaluate the increase in the level of knowledge of final-year nursing students, applying methodological strategies such as problem-based learning (PBL) and film-forum. Methods: 59 nursing students were randomly assigned to two intervention groups (G1 = 31 and G2 = 28), and another 57 were assigned to the control group (without specific classes or workshops on the subject of the study). The intervention consisted of a specific training course on transgender issues (TGSC&W, TransGender Specific Course and Workshop), where the type of meaningful learning strategy used depended on the group (G1 = film-forum and G2 = PBL). The study was carried out at the Faculty of Nursing Nuestra Señora de Candelaria of the Canary Islands Health Service. The randomization was done by blindly choosing a computer-generated code. Results: The main outcome was based on 116 participants, comparing their level of knowledge before and after the workshop. The comparison by pairs shows that there were statistically significant differences (*p* = 0.000) between those undergoing the methodological interventions and the control group. Statistical significance between film-forum and PBL was not obtained (*p* = 1.000): Both methodologies increased the level of knowledge, but there was no significant difference between them. The means for satisfaction with the learning methodology used did not show statistically significant differences. Conclusion: The workshop carried out was highly effective and significant in terms of increasing knowledge. No significant differences were observed in the level of knowledge, or in the degree of satisfaction, between the two methodologies used (PBL and film-forum).

## 1. Introduction

A transgender, or trans, person is someone who does not feel they identify with their sex assigned at birth. Being transgender is still categorized as pathological by the current Diagnostic and Statistical Manual of Mental Illness by the American Psychological Association in Washington D. C., United States [1] (DSM-V). Trans people have a need for both transition-related and non-transition-related healthcare; this is especially true for those who are receiving hormone therapy, adapting their secondary sexual characteristics, or preparing for breast and/or genital reconstructive surgery. However, there is scientific evidence that warns of inequality in the care provided to this group. This fact could be due to the lack of specific training of health professionals [2,3,4,5].

References in the literature show many experiences of educational interventions, outside the official curricula, in gender diversity assistance in healthcare. These courses are for undergraduate and postgraduate students. They have reported successful results [6,7,8] and have in common the use of new learning methodologies to achieve better results.

Surveys by Carabez and Scott (2016) show the need for education and training for nursing practice [9]. Formal training in LGBT matters should be established for key professions, such as nursing, professions where competent care preparation is required [10], and where surveys by Alpert et al. (2017) show that health students were comfortable with training [4].

In Spain, there has been a scarcity of academic or university formative programs in gender diversity and in the care of transgender people. That situation has perpetuated the lack of training in healthcare professionals.

The use of classic pedagogy and educational practices is no longer enough to meet the demands of education [11]. Theorists of education and researchers have, for a long time, proposed active learning strategies. Such strategies increase student participation and promote knowledge retention, critical thinking, and lifelong learning [12]. To achieve this goal, educators must develop innovative teaching strategies to involve students in the philosophical framework of constructivism [13]. In this constructivist framework, there is a place for problem-based learning (PBL) and the film-forum.

PBL has established itself as a valuable educational strategy in the health sciences, proving to be effective not only in the improvement of problem-solving capacity but also in the acquisition of skills such as taking a holistic approach, in self-directed learning [14,15,16,17] and in the promotion of the understanding of the subject in depth [18,19]. With PBL, nursing students use a clinical simulation designed to imitate the real world related to specific healthcare needs and use simulated and programed learning activities to improve their communication, clinical judgment, and confidence [20]. The main components of PBL are a case study, an expert who generates the case, some groups of students (of 8 to 12 students each), and a tutor for each one of the groups. The groups must have a small number of members, to favor optimal collaborative learning [21]. This makes PBL very demanding on resources for the institutions that implement it as part of their teaching strategies.

In the last 20 years, there has been in many countries a growing interest in the use of films in the teaching–learning process in nursing and medicine [22]. Films are a highly attractive instrument and an appropriate teaching method [23], as they are a unique way of encouraging active learning [24], allowing an active use of emotions that provoke reflections, the concretization and development of proposals, and the observation of diverse realities and experiences on the part of the subject as well as his family and social environment [25].

It is possible, therefore, to hypothesize that active learning with these tools (PBL and film-forum) will enable nursing students to learn, synthesize, and apply knowledge when they attend to transgender people who come to the health services. A training program that lasts for three sessions has been designed and analyzed; this program can be incorporated into nursing degree studies and used as a postgraduate course for health science professionals. The objective is to measure the increase in the level of knowledge of the students using these tools and their satisfaction with the program. It also seeks to compare the two methodologies.

## 2. Methods

### 2.1. Design

A prospective study was carried out with nursing students in the academic year 2018/2019 of the Faculty of Nursing Nuestra Señora de Candelaria (EUENSC). EUENSC belongs to the Canary Islands Health Service and is attached to the University of La Laguna and is located on the Spanish island of Tenerife. Ethics approval for the study was obtained through the Committee of Ethics in Medical Research of the University Hospital of the Canary Islands (approval no. CHUNSC_2019_13.ENF19/2019). Sixty new nursing professionals graduate from this university center each year.

A specific course, containing lectures, round tables, and workshops on transgender issues was proposed (TGSC&W, TransGender Specific Course and Workshop). A quasi-experimental intervention study was carried out with two intervention groups and one control group. Each of the intervention groups was given a different type of meaningful learning, and the aim was to measure the variation in the level of knowledge and the satisfaction with the training received. The two interventions used were called film-forum and PBL. Both interventions shared a discussion forum with experts (Supplementary I: TGSC&W leaflet). The word ‘experts’, in this study, refers to all those people with specific knowledge about the topics discussed. Experts are professionals both trans and cisgender persons. Special attention was also given to the specific experiences of trans people and their families. Those people were included within the group of experts. The control group did not receive any type of training or specific activity regarding healthcare for transgender people.

**Figure 1:**
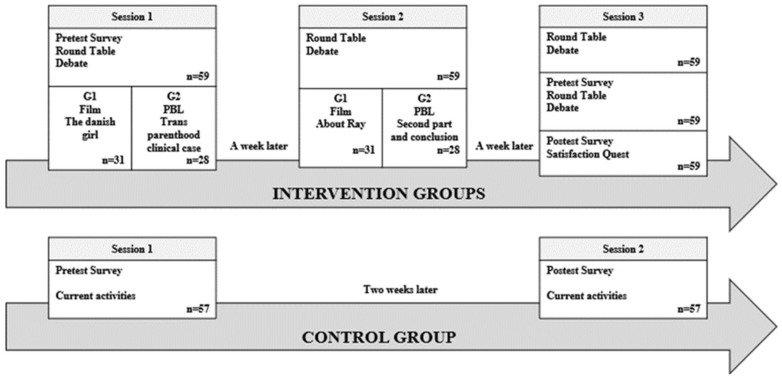
The flow chart of the formative program: TGSC&W sessions.

**Figure 2:**
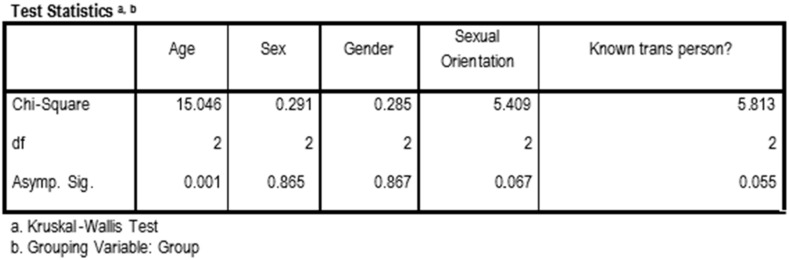
Kruskal–Wallis test. Grouping variable: Group.

The scheme of the three sessions is shown in Figure 1. The number of subjects included in the study was 116, and the students were divided into three groups, two undergoing the methodological intervention (G1 = film-forum and G2 = PBL) and one control group (G3). The Kruskal–Wallis test for independent samples was used for the socio-demographic variables, and for the variable ‘trans-person-known-previously’, and the groups were found to be homogeneous and comparable to each other, with no significant difference except for the age variable, which is logical because third-year students were on average one year younger (Figure 2). The list of characterization variables and measures is shown in Table 1.

### 2.2. Participants

For the intervention groups, fourth-year students were chosen to test the TGSC&W educational tool, which was the main objective of this work. In order to avoid selection bias, all students were invited to participate. This activity did not carry marks or any associated grading.

The control group consisted of third-year students from the same faculty. It was estimated that the study subjects in the intervention and control groups were comparable, in terms of the main variable of the level of knowledge about the care of trans people, and this proved to be the case. This conclusion was reached in light of the fact that there is currently no specific training on this issue within the nursing curriculum. Although no changes were expected in the level of knowledge of the control group, the knowledge level questionnaire was carried out at the beginning of the intervention and was repeated at the end of the intervention period. Control group did not participate in the expert lectures.

The inclusion criteria were third- and fourth-year students with more than 120 approved credits in the European Credit Transfer and Accumulation System (ECTS); there were a total of 120 students, 60 per course. Students who were not enrolled for the entire course, whose number of approved ECTS credits was less than 120, who refused to participate in the study, or who could not attend at least two of the three training sessions were excluded. Students who met all the inclusion criteria and none of the exclusion criteria, and wished to participate, were asked for written consent.

### 2.3. Randomization

For randomization, an opaque bag was used, from which each student chose a piece of paper. On each piece of paper, there was a code, obtained from a computerized random number generator, which the students had to memorize and record in each of the tests for analysis. The randomization of the third- and fourth-year groups was done separately. Each piece of paper had a receipt with the random code, which was kept by the student, and also a brief survey of variables that the student had to fill in and submit.

### 2.4. Intervention (Formative Program)

The educational intervention, TGSC&W, was divided into three sessions over three weeks. Each session was different. Each session was divided into two parts: thematic round table and group-working according to the methodology. The first part was in the form of round table. Transgender people and families and professionals shared vital experiences and knowledge with students. There were lectures on the subject, in which trans and cisgender professionals, trans people, and parents of trans and intersex minors participated. The lectures were conducted by professionals from the fields of medicine, nursing, anthropology, psychology, education, and law so that the students could obtain a comprehensive view of the phenomenon under study. The second part was based on the interaction of students working in groups.

Round Tables: The expert lectures of the first week had an experiential approach. Transgender people themselves and their families told the students about their diverse realities and the problems they had in accessing health services. In the second week, the approach was more professional, with the presence of experts in medicine, nursing, education, and law, who told the students about the present and future of healthcare for transgender people. The third week was a predominantly psychosocial session, with experts from the field of anthropology and psychology participating, giving a more humanized approach to the phenomenon under study.

Group-work: Once the common sessions had ended, each fourth-year group worked according to its methodology separately. The students received complementary audio-visual teaching material in each of the sessions. The film-forum group worked on the past and current history of transgender people, based on the screening of two films, one per session, on the subject: The Danish Girl and About Ray (in their Spanish versions). These films were chosen for their pertinence, and because they showed historical moments and realities or different experiences, both complementary. The PBL group worked on parenthood in transgender people and aspects of this in assisted reproduction, as well as the current state of the situation about this regard.

The adequate acquisition of skills to provide healthcare of the highest possible quality to users is essential for professionals in health sciences. Thus, this intervention on healthcare for transgender people has been carried out as part of the subject called practicum. Practicum is a compulsory subject that gives nursing students specialist knowledge for practicing in different areas. It takes place during the whole of the fourth-year undergraduate program at the University of La Laguna.

### 2.5. Instruments

In order to ensure the reliability and validity of the study, the three groups of students received an explanation of how the measurement method used anonymous tests, and they were told of the importance of the tests being completely and correctly performed. At the beginning, the three groups completed an anonymous questionnaire on the most relevant socio-demographic variables and the Knowledge Questionnaire about Transgender (KQaT). The questionnaire was designed to measure the level of knowledge that students have acquired in the training activity. In this questionnaire, specific questions addressed at the round tables and subsequent discussions with the experts, both trans and cisgender persons, were asked. There was an evaluation exam about a specific activity. The exam was similar to that prepared for any workshop or any subject that is done in the Faculty of Nursing. The exam was carried out by the person responsible for the training activity. In this case there were questions from all the experts involved. The questions were type test, the score from 0 to 10. The increase in the level of knowledge was measured with the difference in score obtained by each individual before and after the educational intervention. Therefore, the KQaT was carried out to assess the previous knowledge level, or the basal notions, of the students. At the end of the third session, all the participants in the intervention and control groups received a post-test questionnaire, KQaT, which was the same as the one that they had filled in during the first week. Control group received the same pre- and post-test at the same time of intervention groups. No significant changes were expected in the control group.

The 30-item KQaT questionnaire was divided into four factors: biological, psychological, social, and legal. The biological factor includes nine items of knowledge about pharmacological treatment and surgery. This factor includes knowledge about the protocol for the care of trans people that is currently in force in the Canary Islands Health Service [26]. This protocol is a clinical guidance for health professionals to assist trans people in accompanying trans people during their transition, to all Canary community-wide, based on the Standards of Care (SOC) established by the World Professional Association for Transgender Health (WPATH) in their manual [27], and adjusted to our environment.

The psychological factor includes eight items on identity and gender-feelings in both adulthood and childhood. The social factor includes seven items that cover the concepts of pathologization and activism, as well as action with transgender minors. The legal factor has six items that include requirements for changing names and genders in legal documents, as well as the current status of legislation in different communities (Supplementary II: KQaT questionnaire).

The questions were closed, multiple choice, questions. Of the four possible responses, only one was true. The decision was made to include some consistency and control questions to check on the congruency of the respondents’ answers. Negative forms were avoided in the wording of the questions, as were ambiguous words. Given the nature of the issue under study, special attention was paid to the use of emotionally charged words such as transphobia, to avoid putting the respondents on the defensive. Each question consisted of a single logical sentence, written briefly.

On the last day of the course, a satisfaction questionnaire was also completed by the intervention groups. This questionnaire asked the opinions of the students about the formative plan itself. A Likert scale was used [1 (strongly disagree) to 10 (strongly agree)] to respond to 25 items pertaining to the course. The questions were about whether the respondents feel that their knowledge, skills, and abilities on the subject had improved, whether individual or group learning capacity was encouraged, the degree of comfort with the methodology used, and whether the teacher–student interaction could have been improved, among other issues.

### 2.6. Analysis

Data analysis was performed using SPSS/WIN19.0. The main variables of the study were: the level of knowledge, measured with the score obtained in the knowledge test (KQaT), and the satisfaction with the training received, measured with the score obtained in the satisfaction test. The teaching methodology used (film-forum or PBL) was also included. In addition, socio-demographic variables were considered, such as age, gender, gender identity, sexual orientation, and the question “do you know any transgender person?” (where the understanding of the phrase “to know” was that the person had some type of relationship: friends or acquaintances, classmates, family).

The characteristics of the control group and the two intervention groups were controlled and analyzed by a hypothesis test at the beginning of the study.

The score of KQaT was 0 to 10. The answer of the questions was in test type. There was no penalty for failed answers. A specific cut-off value was not used to pass or suspend the test. The relationship sought was the difference between the level of knowledge obtained by the different groups, before and after the learning activity.

The content validity of the KQaT questionnaire was assessed by nine experts (two professors, one physician, two nurses, a psychologist, a lawyer, an anthropologist, and a biologist). The experts met several times to prepare the final questionnaire, which was based on the topics that they themselves would discuss with the students in the three workshop sessions.

The data from the questionnaires were processed separately in the three groups and the means and standard deviations are shown in the tables. The main outcome variables were analysed as follows: for the analysis of the level of knowledge acquired from the workshop over the three sessions (TGSC&W), an ANCOVA was carried out. The prior knowledge (the score obtained in the pretest) was taken as a covariate, on the basis that a student’s degree of subsequent knowledge may be mediated by his or her degree of prior knowledge. With the ANCOVA this factor is eliminated, and more accurate values are obtained. To test the satisfaction with the methodology, a Student *t*-test was performed. An analysis of the reliability, using Cronbach’s alpha coefficient, was performed.

### 2.7. Ethics Approval and Consent to Participate

This study was approved by the Institutional Review Board of the Ethics Committee of the University Hospital La Candelaria, Canary Islands Public Health Service (approval no. CHUNSC_2019_13.ENF17/2019). Written informed consent was obtained from all participating students.

## 3. Results

**Figure 3:**
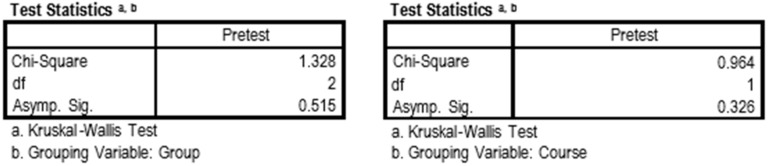
Kruskal–Wallis test. Grouping variable: Group and Course.

The main outcome variable was the knowledge obtained after the TGSC&W sessions. This variable may be influenced by prior knowledge. Therefore, a homogeneity study of this last variable in the groups was carried out. Whether the Course (third or fourth year) or the Group (G1, G2, or G3) was used as a grouping variable, the samples were comparable at the beginning for the previous knowledge variable (*p* > 0.05), considering the three groups as equal (Figure 3).

### 3.1. Level of Knowledge Acquired

**Figure 4:**
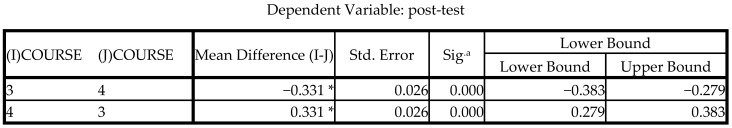

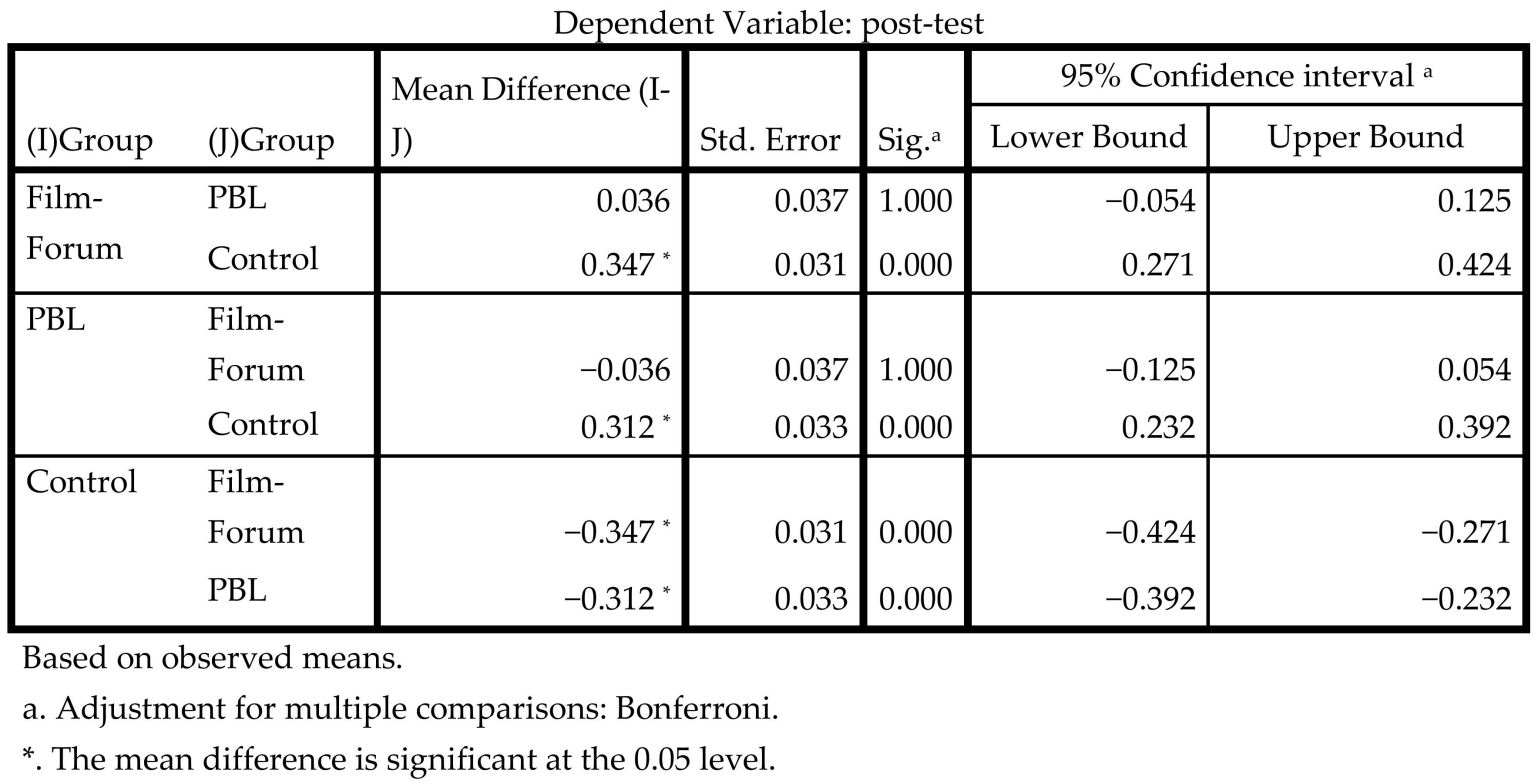
Comparison by course and group.

The comparison by course showed that after the intervention there were statistically significant differences in the level of knowledge of the fourth-year students compared to the third-year students (*p* = 0.000). By making a comparison by groups, the different interventions carried out were considered. The pairwise comparison showed that there were statistically significant differences (*p* = 0.000) between the two methodological interventions used with respect to the control group. However, no significance could be shown (*p* = 1.000) between film-forum and PBL. Both methodologies increased the level of knowledge, but there was no significant difference between them (Figure 4).

**Figure 5:**
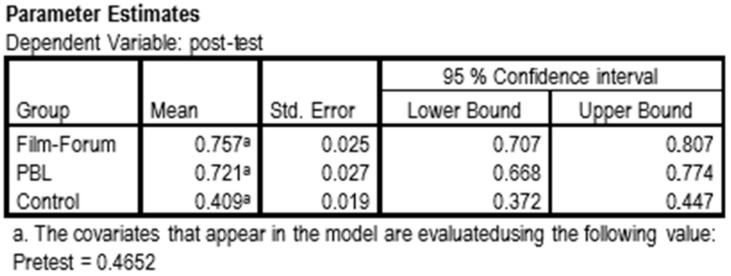
Knowledge measures obtained based on the reference.

Once the possible effect of the previous training has been controlled for, the calculation of estimates gives a value of 0.4652 as a pretest reference value. Taking that value as a reference, the students in the control group G3 had not obtained any more knowledge in the post-test (mean = 0.409), while students in both the G1 and G2 groups had raised their average knowledge score (G1 = 0.757 and G2 = 0.721) (Figure 5).

The improvement in the level of correct answers to the KQaT questionnaire, comparing the previous and subsequent phases, was significant and homogeneous in all the items separately and grouped by factors. 

### 3.2. p Values with Chi-Square Test 

Table 2 shows the degree of knowledge obtained in the different factors, according to the number of valid participants (the 59 students of the intervention groups who answered the KQaT questionnaire both before and after the intervention).

### 3.3. Level of Satisfaction with the Methodology Used

The results of the means for satisfaction with the methodology were the following: For the G1 of film-forum, it was 8.04 out of 10, while in the G2 it was 8.45 out of 10. There is no statistical significance between these figures. Reliability analysis of the items showed that Cronbach’s alpha was 0.81.

## 4. Discussion

The study that the authors have presented in this paper is the first course in transgender studies for undergraduate nurses in Tenerife, Canary Islands (Spain). The students had little prior knowledge of transgender issues. Therefore, for nursing students, the importance of this course was not merely to gain knowledge of the techniques and protocols applicable to this population. It essentially requires students to treat the person holistically. Moreover, it offers the first opportunity for prospective nurses to develop appropriate advice or plans for transgender people in need of health assistance in the future.

The choice of the subject to be discussed was important. There are studies carried out by different authors that conclude that, on the one hand, transgender people feel that they are not adequately cared for by health personnel and, on the other hand, that health professionals have a training deficit in the health needs of transgender people [2,3,4,5]. Surveys such as Jones (2019) [28] and Smith et al. (2014) [29] show that patients rate their health care experience as positive, if previously such staff have been specially trained.

Through a brief educational intervention for training in gender diversity, TGSC&W, it has been possible to verify an increase in knowledge among nursing students about the phenomenon under study. By controlling the marginal means (that is, the means remaining after removing the effect of the covariate ‘level of previous knowledge’), it has been possible to confirm the increase of knowledge once the possible effect of previous knowledge on later knowledge has been controlled In this study, the reference value for prior knowledge was 0.4652. These data allow the conclusion to be drawn that the students in the control group did not obtain any more knowledge at the time of the post-test (mean = 0.409); in fact, their starting knowledge was even lower than estimated. However, students in both G1 and G2 increased their average knowledge (G1 = 0.757 and G2 = 0.721). All this shows that the gain in knowledge was greater than expected. These data spoke of the usefulness of the model that was designed to increase the knowledge of nursing students. In general, ad hoc programs that are designed to cover specific knowledge gaps are usually quite effective [30,31]. In the next stage, it would be necessary to incorporate this type of training into the official study plans.

Another relevant finding that was shown by this intervention was that both methodologies were equally effective in the acquisition of knowledge and gave a great difference over the control group. This has also been found in similar studies where the discrepancy between tests before and after an intervention [20,32,33,34] was studied with one or another methodology. This is in contrast with the study conducted by Carpenter et al. which concluded that there was no evidence of an improvement in students’ understanding and no gain in knowledge [35]. Despite these teaching methodologies being well-developed, this is the first study to be carried out comparing the two significant learning procedures [36]. As previously mentioned, the PBL methodology is more demanding in teaching and infrastructure resources, since it works with small groups that each requires at least the presence of a teacher–tutor, and separate spaces to work in [21]. The results of this study showed that the two methodologies are comparable in terms of knowledge acquisition and satisfaction with the teaching methodology. Therefore, and given the lower expense it requires, the film-forum can be a very important alternative for this type of significant learning.

The average satisfaction with both intervention methodologies was high, with averages of 8.04 (film-forum) and 8.45 (PBL) out of 10. This result agrees with the findings of Kim et al., who found an average of 4.18 out of 5 [20], and of Lin et al., with an average of 7.64 out of 10 [37].

The main objective of the KQaT questionnaire was to measure the empirical variable of knowledge level after attending the workshop, with concrete questions capable of eliciting reliable, valid, and quantifiable answers. The division of the KQaT questionnaire into factors made it possible to demonstrate the improvement in the different areas of knowledge that must be shown by a senior student (or a health professional) if he/she is to care for the patient in a comprehensive manner. Analyzing each factor explored, the area of biology was where there was the greatest degree of knowledge gain (accurate pre: 251 vs. accurate post: 394. Difference: 143. *p* = 0.000), and there was less knowledge gained in the legal area (accurate pre: 137 vs. accurate post: 238. Difference: 101. *p* = 0.000). An explanation could lie in the fact that the intervention was carried out with nursing students who were studying for a degree in health sciences and were familiar with the terms used and were trained in acquiring new knowledge related to this science. By contrast, the legal aspects, endowed with a language of their own and with a purpose far from that of a nursing professional, made the internalization of knowledge related to this factor difficult for a health science student. Even so, it is worth noting that there was a gain in knowledge for all the factors explored.

The main focus of this study is the short-term benefits for the nursing students. However, the benefits of this training in the long-term could be very powerful. They are the provision of trained professionals in response to the calls from transgender people seen in the literature and their affirmations of how this improved their likelihood of seeking and maintaining medical care on other health issues [28,29]. In the long-term, this destigmatization work can save the lives of trans patients, by ensuring they can seek medical care not only on gender issues but for their everyday screenings or more serious healthcare needs. The literature discusses their avoidance of healthcare professionals [28,29]. The work presented in this study could be a powerful solution and it is appropriate to note this long-term likely outcome as affirmed by the literature.

## 5. Difficulties and Limitations of the Study

The small sample size and the very specific profile (nursing students in the fourth year of their degree) mean that it is not possible to extrapolate the results. When working with a small and opportunistic sample, its representativeness and the power of extrapolation can be analyzed a posteriori. However, the objective was to design a brief intervention that can be replicated in small groups, so the results can be expected to be applicable to similar groups of students and professionals.

Groups in the EUENSC usually coexist and exchange experiences during their training. This could mean that there was contamination from one group to the other, which would give rise to a problem of validity. To minimize the impact of this possible contamination, two measures were taken. The first was to conduct the sessions when the students were exclusively in clinical practice, that is when the 120 students were scattered throughout the hospital units, outpatient clinics, ambulances, and centers of specialized care as part of their course. During the period of this study, the students did not coincide as a group for any activity. The second measure was to differentiate, to the maximum possible degree, the subject matter on which the students worked. This meant that, if they were to contact each other, the knowledge acquired in one group was not useful in the other group. Thus, the film-forum group worked on the past and current history of transgender issues and the PBL group worked on parenthood in transgender people and aspects of assisted reproduction as well as the current state of legislation on this matter.

There was also the prospect that some students were from the population of interest and therefore would have knowledge and insight and were not identified as such. 

As a limitation, the authors applied non-standardized instruments to measure learning progress.

## 6. Conclusions

Our intervention study has been proved to be highly effective and significant in terms of increasing students’ knowledge, with both the methodologies used. To choose between them, it would be necessary to assess the different resources required to carry them out.

Given the good results from the TGSC&W intervention, this could be an alternative for the formative proposal in the health sciences curriculum. Besides, this formative intervention could be extrapolated to professionals themselves who have no training in gender diversity, contributing unequivocally to improvements in the care provided to transgender people.

Spain has laws ranging from the right to change gender and name in legal documents, such as anti-discrimination laws. But it does not have a trans state law, in addition to the fact that there are protocols that require education in gender and sexual diversity, these protocols do not exist for higher or university education. Our study could be replicated in countries with a situation similar to ours.

## Figures and Tables

**Table 1:** Variables and measures.

		4° Grade		3° Grade
		G1 (*n* = 31) Film-Forum	G2 (*n* = 28) PBL	G3 (*n* = 57) Control Group
Age	Mean (dt)	22.97 (4.086)	22.71 (3.619)	22.11 (4.861)
	Minimum	20	20	19
	Maximum	40	33	48
Biological Gender	Male	7	8	14
	Female	24	20	43
Gender Identity	Male	7	8	15
	Female	24	20	42
	Gender Fluid	-	-	-
	Agender	-	-	-
Sexual Orientation	Heterosexual	24	21	53
	Homosexual	3	3	2
	Bisexual	4	4	2
	Pansexual	-	-	-
	Asexual	-	-	-
Know transgender person?	No	20	20	28
	Yes	8	8	29
	Not sure	3	-	-

**Table 2:** Improvement of the accuracy of the KQaT test responses by factors, before and after the intervention (*n* = 59).

FACTORS	Pretest Intervention Groups *n*	Post-Test Intervention Groups *n*	Difference	*p* *
Biological (9 questions) (Questions *n*° 10, 11, 12, 13, 17, 21, 27, 28, 30)	Accurate = 251	Accurate = 394	143	0.000
Psycological (8 questions) (Questions *n*° 1, 18, 19, 20, 22, 23, 24, 26)	Accurate = 307	Accurate = 436	129	0.000
Social (7 questions) (Questions *n*° 4, 5, 6, 7, 8, 9, 29)	Accurate = 140	Accurate = 248	108	0.000
Legal (6 questions) (Questions *n*° 2, 3, 14, 15, 16, 25)	Accurate = 137	Accurate = 238	101	0.000

## Supplementary Materials

The following are available online at https://www.mdpi.com/1660-4601/16/17/3205/s1, Supplementary I: TGSC&W leaflet, Supplementary II: KQaT questionnaire.
