# Supplementary material for: Impact of a Formative Program on Transgender Healthcare for Nursing Students and Health Professionals. Quasi-Experimental Intervention Study
Source: Int J Environ Res Public Health. 2019 Sep 2;16(17):3205. doi: 10.3390/ijerph16173205 (PMC6747280; doi:10.3390/ijerph16173205)
Supplement: Supplementary file 1 [file ijerph-16-03205-s001.zip › Supplementary I TGSC&W leaflet.pdf]

Este proyecto trata de mejorar la atención que prestamos a las personas trans

Para ello pretendemos trabajar en la formación de los nuevos profesionales de forma multidisciplinar.

El acercamiento y el conocimiento a las personas y a la realidad trans reduce barreras y aumenta la calidad de los cuidados en salud que les prestamos a nuestros usuarios.

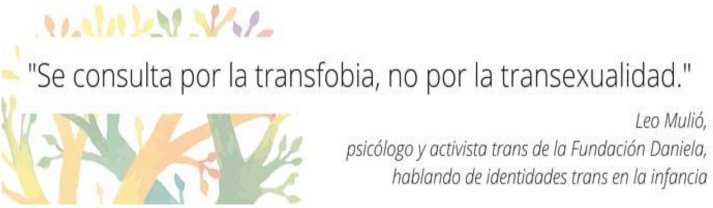

"Sexo es lo que se ve, género es lo que se siente. La armonía entre ambos es esencial para la felicidad del ser humano."

Harry Benjamín,1976

Escuela Universitaria de Enfermería Ntra. Sra. de Candelaria

Universidad de La Laguna

Servicio Canario de la Salud

Gobierno de Canarias

Colaboran: TransGirls Chrysallis

Atención Trans-competente centrada en la persona

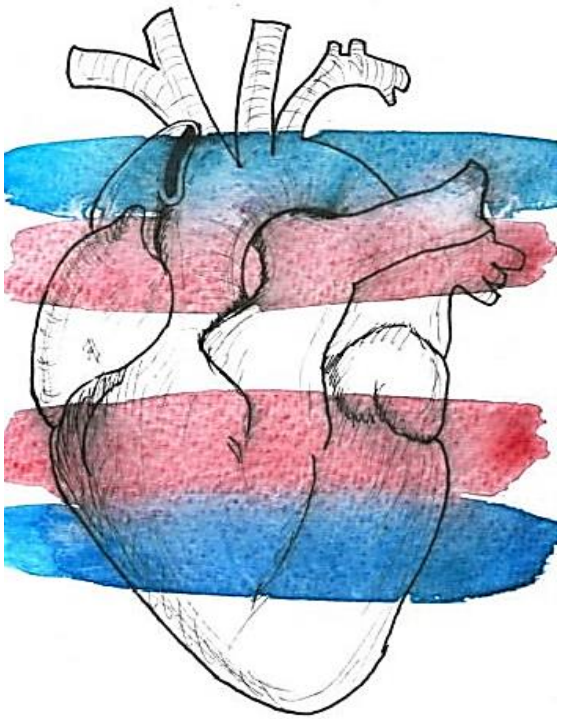

6, 13 y 20 de noviembre 2018
EUENSC- Practicum

6 de Noviembre

- *8:00-8:30h Presentación y explicación de la actividad*
- *8:30-9:00h Apertura del Seminario-Taller por parte del Sr. Jesús Delgado Santana, Director Gerente del Hospital Nstra. Sra. de Candelaria*
- *9:00-9:15h Exploración nivel de conocimientos*
- *9:15-10:30h Mesa redonda*
- *10:30-11:00h Descanso*
- *11:00-13:30h Actividad según grupo*
- *13:30-14:00h Debate en grupo*

13 de Noviembre

- *8:30-10:00h Mesa redonda*
- *10:00-10:30h Descanso*
- *10:30-13:00h Trabajo en grupo*
- *13:00-13:30h Debate en grupo*

OMS

El 18 de junio de 2018, saca la TRANSEXUALIDAD de la lista de ENFERMEDADES MENTALES.

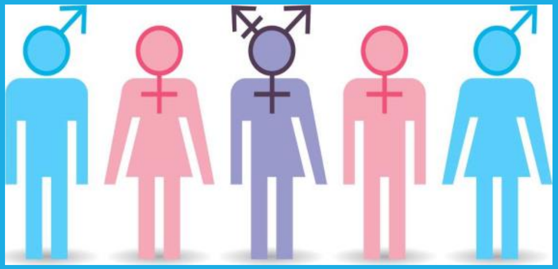

20 de Noviembre

- *8:30-10:00h Trabajo en grupo*
- *10:00-10:15h Cuestionario de satisfacción con la herramienta*
- *10:15-10:45h Descanso*
- *10:45-11:30h Exposición y debate sobre el trabajo realizado*
- 11:30-13:30h *Mesa redonda*
- 13:30-13:45h *Exploración nivel de conocimientos*
- 13:45h Cierre de la actividad por la Dra. Ana Isabel Jimenez Avizanda, Vicerrectora de Docencia de la ULL.

Ponentes invitados

Profesionales de:

Enfermería:

Doctorando Jesús M. García Acosta

Antropología

Dra. M. Elisa de Castro Peraza

Psicología

Dra. Lilisbeth Perestelo Pérez

Medicina

Dra. Nieves D. Lorenzo Rocha

Derecho

Sra. Carla Cardona Llabrés

Educación

Doctorando Alfredo Fernández Martínez

Asociaciones Colaboradoras:

Transboys: Sr. Eduardo Gestido Castilla

Transgirls: Sra. Yuli Pérez Yumar

Sección Familias Transboys y Transgirls: Sra Clara Montesdeoca Castilla

Chrysallis Tenerife: Dra. Olga Díez Fernández
