# Supplementary material for: Impact of a Formative Program on Transgender Healthcare for Nursing Students and Health Professionals. Quasi-Experimental Intervention Study
Source: Int J Environ Res Public Health. 2019 Sep 2;16(17):3205. doi: 10.3390/ijerph16173205 (PMC6747280; doi:10.3390/ijerph16173205)
Supplement: Supplementary file 1 [file ijerph-16-03205-s001.zip › Supplementary II KQaT questionnaire.pdf]

CUESTIONARIO DE CONOCIMIENTOS SOBRE DIVERSIDAD DE GÉNERO (KQaT) (SPANISH VERSION)

1. Para reconocer la transexualidad en la infancia las familias deben:
- a. Recurrir a un psiquiatra que diagnostique la transexualidad
- b. Recurrir a un psicólogo que emita un informe
- c. Escuchar al menor y respetar su identidad, sin censurar
- d. Corregir toda expresión de género (juegos, ropa, amistades...) que vaya en contra de su sexo registral

2. Se entiende por sexo registral:
- a. El que se establece a partir de las características fisiológicas de una persona.
- b. El que se indica en la partida de nacimiento según figura en el registro civil.
- c. El que la persona comunica libremente ante la sociedad
- d. El que se indica mediante certificado médico.

3. En la ley en vigor 3/ 2007, del 15 de marzo, reguladora de la rectificación registral en cuestión relativa al sexo de las personas:
- a. No se contempla la transexualidad en menores.
- b. No se contempla la transexualidad en menores ni en residentes extranjeros.
- c. No se permite el cambio de sexo sin operación genital
- d. Las personas pueden solicitar la corrección de la mención registral de sexo sin ningún requisito médico.

4. La patologización de la transexualidad conlleva una serie de consecuencias, como son:
- a. Violaciones de los derechos humanos
- b. Anomalías o malformaciones
- c. Limitación importante de derechos de acceso a los recursos públicos
- d. Todas son correctas

5. ¿El activismo por la despatologización de la intersexualidad busca fundamentalmente?
- a. Defender el derecho a la diversidad corporal
- b. Defender el derecho a la diversidad de género
- c. Defender el derecho a la diversidad sexual
- d. Defender el derecho a la diversidad cultural

6. Los principios de Yogyakarta…
- a. Enuncian la aplicación de la normativa de la ONU sobre derechos de las personas transexuales, en el ámbito de los países asiáticos y del pacífico
- b. Son un método de enunciar los derechos de las personas intersexuales
- c. Son unas conclusiones extraídas del conflicto de la cafetería Comptom´s, inicio de la revuelta por los derechos de los transexuales
- d. Son la aplicación de la carta de derechos humanos a la diversidad sexual, corporal y de género

7. El protocolo para el acompañamiento al alumnado trans y la atención a la diversidad de género en los centros educativos de Canarias se pone en marcha:
- a. Únicamente si la situación es comunicada por las progenitoras, los progenitores o representantes legales del alumno o alumna trans al centro educativo.
- b. Si es comunicada por cualquier miembro de la comunidad educativa.
- c. Exclusivamente si es comunicado por el propio alumnado trans, para preservar su intimidad.
- d. Si es comunicada la Inspección Educativa y esta da la orden de aplicar el protocolo.

8. **Una vez activado el protocolo para el acompañamiento al alumnado trans y la atención a la diversidad de género en los centros educativos de Canarias el centro:**
    a. Permitirá el acceso a aseos y vestuarios acordes a su identidad de género.
    b. Podrá obligar al alumnado a vestir con el "uniforme escolar" propuesto por el Consejo Escolar hasta que no tenga modificado su nombre en el Registro Civil.
    c. Pondrá obligatoriamente las horas de la asignatura de Educación Física a última hora del día en los cursos con alumnado trans.
    d. Acondicionará un nuevo aseo diferente a los dos existentes, pudiendo ser construido de forma anexa al edificio principal si es necesario, para uso exclusivo al alumnado trans.

9. **Dentro de las actuaciones de sensibilización, formación y adecuación curricular para la inclusión de la diversidad sexual y de género, los centros educativos podrán realizar actividades complementarias y/o extraescolares aprovechando fechas conmemorativas como pueden ser:**
    a. 31 de marzo: "Día Internacional de la Visibilidad Trans".
    b. 24 de mayo: "Día Internacional contra la Lesbofobia, Homofobia, Transfobia, Bifobia e Interfobia".
    c. 18 de junio: "Día del Orgullo Lésbico, Homosexual, Trans, Intersexual, e Identidades No Binarias".
    d. 24 de octubre: "Día de la Visibilidad Intersex".

10. **El tratamiento hormonal se podrá iniciar:**
    a. Antes de desarrollo de los caracteres sexuales secundarios
    b. Siempre debe existir experiencia de vida real documentada
    c. Cuando la persona transexual decida operarse
    d. Todas son falsas

11. **Los efectos del tratamiento hormonal, es falso que:**
    a. En su mayoría son reversibles
    b. En transexuales de hombre a mujer hay respuesta parcial en la eliminación del vello facial
    c. En transexuales de mujer a hombre, el tamaño mamario disminuye
    d. Las primeras modificaciones corporales aparecen generalmente antes de los 3 meses

12. **Como efectos adversos del tratamiento hormonal destacan:**
    a. El tromboembolismo venoso en caso de terapia hormonal androgénica
    b. Resistencia a la acción de la insulina con estrógenos
    c. Con el uso de estrógenos, el cáncer de mama
    d. Con el uso de testosterona, riesgo de moderado a alto de migraña intensa

13. **Sobre el tratamiento quirúrgico de reasignación:**
    a. Precisa de un año de tratamiento hormonal continuo, salvo que existan contraindicación médica para dicho tratamiento
    b. Debe conocer los distintos tipos de intervención, costes, duración de la hospitalización, posibles complicaciones y rehabilitación tras la cirugía
    c. Debe firmar el consentimiento informado
    d. Todas son correctas

14. **Para efectuar un cambio de nombre en el Registro, es necesario:**
    a. La existencia previa de un trastorno de personalidad diagnosticado
    b. Declaración jurada del sexo reclamado por parte del solicitante
    c. Es condición necesaria la existencia previa de cirugía de reasignación sexual
    d. La existencia previa de un diagnóstico de disforia de género

15. **Una vez efectuado el cambio de nombre registral, este hecho comportará:**
    a. La obligación de tramitación de un nuevo DNI acorde a la inscripción registral efectuada, con diferente número de DNI.
    b. No comporta obligación de tramitación de nuevo DNI, basta con la mera notificación
    c. La obligación de tramitación de un nuevo DNI, conservando el número anterior, acorde con la inscripción registral efectuada
    d. La notificación desde el Registro correspondiente, a todos los organismos, con la nueva inscripción registral detallada

16. **Ley 2/2014, de 8 de julio, integral para la no discriminación por motivos de identidad de género y reconocimiento de los derechos de las personas transexuales, es una Ley de aplicación en la Comunidad Autónoma de:**
    a. Madrid
    b. Cataluña
    c. Andalucía
    d. Es de aplicación a nivel nacional

17. **El sexo biológico es:**
    a. Aquel sexo con el que se nace y que viene determinado por los genitales.
    b. El sexo de nacimiento y que viene determinado genéticamente.
    c. El sexo de nacimiento que viene dado por los genes siendo masculino o femenino.
    d. El sexo de nacimiento que viene determinado por los genes siendo macho o hembra.

18. **El sentimiento de pertenencia a un género u otro es conocido como:**
    a. Pansexualidad.
    b. Transgenerismo.
    c. Identidad.
    d. Homosexualidad.

19. **Si una persona a la que se le asignó un determinado sexo al nacimiento tiene un sentimiento de pertenencia al otro género. Hablamos de:**
    a. Pansexualidad.
    b. Cisexualidad.
    c. Travestismo.
    d. Transexualidad.

20. **El sentimiento de pertenencia a un determinado sexo:**
    a. Sexo sentido.
    b. Género sentido.
    c. Sexo perteneciente.
    d. Género pertinente.

21. **¿Qué es la masculinización del tórax?**
    a. Ese procedimiento quirúrgico aún no está implantado en España, sí se hace en el resto de la Unión Europea.
    b. Es un procedimiento quirúrgico que se le realiza a las mujeres tras padecer cáncer de mama, y que resulta en un pecho de apariencia masculina.
    c. Es un procedimiento quirúrgico consistente en una mastectomía bilateral subcutánea con extirpación del tejido mamario, y reducción del tamaño de las areolas y los pezones conforme al patrón masculino.

d. Es un procedimiento quirúrgico que se basa en una mastectomía bilateral subcutánea para reducir el volumen del pecho, dejando tejido mamario y cicatriz visible y en la que la mayoría de veces no es necesario usar un injerto areola-pezón.

22. ¿Qué entiendes por un hombre transexual?

a. Una persona a la que se le ha asignado el sexo masculino al nacer y se siente mujer.
b. Una chica que quiere ser chico.
c. Una persona a la que se le ha asignado el sexo masculino al nacer y se opera para ser mujer.
d. Una persona a la que se le ha asignado el sexo femenino al nacer y se siente hombre.

23. El cambio o adecuación de caracteres secundarios hacia el sexo sentido se conoce como:

a. Test de vida real.
b. Tránsito.
c. Transgenerismo.
d. Expresión del género.

24. ¿Cuándo un niño tiene conciencia sobre su género?

a. 3-4 años.
b. 5-6 años.
c. 7-11 años.
d. 9-14 años.

25. Señale la opción que considere correcta:

a. Todas las comunidades autónomas siguen un protocolo de atención sanitaria a personas trans establecido por la OMS mediante guías de recomendación de práctica clínica.
b. Únicamente algunas comunidades tienen protocolos de atención sanitaria a personas trans, entre ellas Canarias.
c. Como es algo de nueva emergencia en la sociedad, aún no existe un protocolo de actuación.
d. Únicamente existe en Canarias un protocolo de acompañamiento al alumnado trans, en los centros escolares.

26. Si una persona nace con un sexo biológico de mujer, pero se identifica con el género masculino y se siente atraído sexualmente por otras mujeres hablamos de:

a. Una mujer homosexual.
b. Una mujer heterosexual.
c. Un hombre homosexual.
d. Un hombre heterosexual.

27. ¿Hay que someter a cirugías a los bebés intersexuales?

a. No, nunca, estos procedimientos son irreversibles y da como resultado un cuerpo desprovisto de sensibilidad sexual en la mayoría de los casos.
b. Sí, siempre que sea necesario una vez valorados por cirugía pediátrica.
c. Depende de lo que decidan los padres.
d. Depende de lo que opinen los médicos y los padres, una vez llegue el resultado del cariotipo.

28. ¿Es los mismo ser intersexual que ser transexual?

a. Sí, depende de los gustos sexuales.
b. La intersexualidad es una forma más de diversidad de género.
c. La intersexualidad no es una parte de la diversidad de género, porque la intersexualidad no es acerca del género, ni tiene que ver con una transición.
d. Según el tamaño de los genitales con los que se nazca será una cosa o la otra

29. ¿Qué crees que es una persona cisexual?

a. Una persona a la que se le asigna un sexo al nacer según sus genitales.
b. Una persona a la que se le asigna un sexo al nacer según sus genitales y se siente conforme con ese sexo asignado.
c. Una persona a la que se le asigna un sexo al nacer según sus genitales y no se siente conforme con ese sexo asignado.
d. Una persona que se realiza una reasignación sexual mediante procedimientos quirúrgicos.

30. ¿En qué se diferencian los conceptos intersexual y hermafrodita?

a. Es lo mismo, antes se llamaba hermafroditas y ahora intersexuales.
b. La diferencia radica en la falta de genitales de la persona intersexual.
c. La diferencia radica en que las personas con genitales intersexuales no pueden auto fecundarse.
d. La diferencia radica en el tamaño de los genitales, según sea de un tamaño menor será intersexual y de un tamaño mayor será considerado hermafroditismo.

GRACIAS POR TU COLABORACIÓN

KNOWLEDGE QUESTIONNAIRE ABOUT TRANSGENDER (KQaT) (ENGLISH VERSION)

1. To recognize transgender or transsexuality in childhood, families must:
- a. Resort to a psychiatrist who diagnoses transsexuality
- b. Resort to a psychologist who issues a report
- c. Listen to the child and respect their identity, without censoring
- d. Correct any gender expression (games, clothes, friendships ...) that goes against their sex assigned at birth

2. It is understood by sex of registration:
- a. The one which is established from the physiological characteristics of a person.
- b. The one indicated in the birth certificate as it appears in the civil registry.
- c. The one that the person communicates freely to society
- d. The one which is indicated by a medical certificate.

3. In the law in force 3/2007, of March 15, regulating the registration rectification in questions regarding the sex of persons:
- a. Transgender or transsexuality in minors is not contemplated.
- b. Transsexuality in minors or foreign residents is not contemplated.
- c. Sex change is not allowed without genital surgery
- d. People can request the correction of the registration of sex without any medical requirement.

4. The pathologisation of transsexuality carries a series of consequences, such as:
- a. Human rights violations
- b. Anomalies or malformations
- c. Important limitation of access to public resources
- d. All answers are correct

5. Activism for the depathologisation of intersexuality fundamentally seeks
- a. Defence of the right to body diversity
- b. Defence of the right to gender diversity
- c. Defence of the right to sexual diversity
- d. Defence of the right to cultural diversity

6. The principles of Yogyakarta ...
- a. They enunciate the application of the UN regulations on the rights of transgender people, in the field of Asian and Pacific countries
- b. They are a method of enunciating the rights of intersex people
- c. They are conclusions drawn from the conflict of the Comptom´s cafe, the beginning of the revolt for the rights of transsexuals
- d. They are the application of the human rights charter to sexual, bodily and gender diversity

7. The protocol for the accompaniment of trans students and the attention to gender diversity in the educational centres of the Canary Islands is launched:

a. Only if the situation is communicated by the parents or legal representatives of the trans student to the educational centre.
b. If it is communicated by any member of the educational community.
c. Exclusively if it is communicated by trans students themselves, to preserve their privacy.
d. If the Educational Inspection is communicated and it gives the order to apply the protocol.

8. Once the protocol for the accompaniment of trans students and the attention to gender diversity in the schools of the Canary Islands has been activated, the centre:

a. Will allow access to toilets and changing rooms according to their gender identity.
b. May force students to wear the "school uniform" proposed by the Council School until the student has modified your name in the Civil Registry.
c. Will obligatorily put the hours of the Physical Education subject at the last hour of the day in courses with trans students.
d. Will condition a new toilet different from the two existing ones, that can be built adjacent to the main building if necessary, for the exclusive use of trans students.

9. Within the actions of sensitization, training and curricular adaptation for the inclusion of sexual and gender diversity, educational centres may carry out complementary and / or extracurricular activities; taking advantage of commemorative dates such as:

a. March 31: "International Day of Trans Visibility".
b. May 24: "International Day against Lesbophobia, Homophobia, Transphobia, Biphobia and Interphobia".
c. June 18: "Lesbian, Gay, Trans, Intersexual, and Non-Binary Identity Pride Day".
d. October 24: "Intersex Visibility Day".

10. Hormonal treatment should be initiated:

a. Before the development of secondary sexual characteristics
b. There should always be documented in real-life experience report (according to the protocol)
c. When the trans person decides to have surgery
d. They are all false

11. About the effects of hormonal treatment, it is false that:

a. They are mostly reversible
b. In male to female trans, there is a partial response in facial hair removal
c. In female to male trans, breast size decreases
d. The first body modifications usually appear before three months

12. The adverse effects of hormonal treatment include:

a. Venous thromboembolism in case of androgenic hormone therapy
b. Resistance to the action of insulin with estrogen
c. With the use of estrogen, breast cancer
d. With the use of testosterone, moderate to high risk of severe migraine

13. About surgical reassignment treatment:

a. It takes one year of continuous hormonal treatment unless there are medical contraindications for such treatment.

b. The person should know the different types of intervention, costs, length of hospitalization, possible complications and rehabilitation after surgery
c. The person must sign the informed consent
d. All answers are correct

14. To make a change of the person's name in the Registry, it is necessary:
a. The prior existence of a diagnosed personality disorder
b. Affidavit of the sex claimed by the applicant
c. The prior existence of sexual reassignment surgery is a necessary condition
d. The previous existence of a diagnosis of gender dysphoria

15. Once the change in the registration-name has been made, this will entail:
a. The obligation to process a new ID, according to the registration made, with a different ID number.
b. There is no obligation to process a new ID, just the mere notification
c. The obligation to process a new ID, keeping the previous number, in accordance with the registration made
d. The notification from the corresponding Registry, to all organizations, with the new detailed registration

16. Law 2/2014, of July 8, integral for non-discrimination based on gender identity and recognition of the rights of transsexual persons, is a law applicable to the Autonomous Community of:
a. Madrid
b. Catalonia
c. Andalusia
d. It is applicable nationwide

17. The biological sex is:
a. That sex with which one is born and that is determined by the genitals.
b. The sex of birth and that is determined genetically.
c. The sex of birth that is given by the genes being man or woman.
d. The sex of birth that is determined by the genes being male or female.

18. The feeling of belonging to one gender or another is known as:
a. Pansexuality
b. Transgenerism
c. Identity.
d. Homosexuality.

19. If a person to whom a certain sex was assigned at birth has a feeling of belonging to the other gender. We talk about:
a. Pansexuality
b. Cissexuality
c. Transvestism.
d. Transgender or transsexuality

20. The feeling of belonging to a certain sex is knowing as:
a. Sex felt.
b. Gender felt.
c. Belonged sex.

d. Relevant gender

21. What is the surgery for masculinization of the thorax?

a. This surgical procedure is not yet implemented in Spain, it is done in the rest of the European Union.

b. It is a surgical procedure that is performed on women after suffering from breast cancer, and that results in a male-looking breast.

c. It is a surgical procedure consisting of a bilateral subcutaneous mastectomy with removal of breast tissue, and reduction of the size of the areolas and nipples according to the male pattern.

d. It is a surgical procedure that is based on a bilateral subcutaneous mastectomy to reduce breast volume, leaving breast tissue and scar visible and in which most of the time it is not necessary to use an areola-nipple graft.

22. What do you understand by a trans man?

a. A person who has been assigned the male sex at birth and feels female.

b. A girl who wants to be a boy.

c. A person who has been assigned the male sex at birth and is operated to be a woman.

d. A person who has been assigned the female sex at birth and feels male.

23. The change or adaptation of secondary characters towards felt sex is known as:

a. Real life test

b. Transit.

c. Transgenerism

d. Gender expression

24. When is a child aware of his gender?

a. 3-4 years

b. 5-6 years.

c. 7-11 years.

d. 9-14 years.

25. Indicate the option you consider correct:

a. All Autonomous Communities follow a protocol for health care for transgender persons established by the WHO through clinical practice recommendation guides.

b. Only some communities have health care protocols for trans people, including the Canary Islands.

c. As it is something of a new emergency in society, there is still no action protocol.

d. There is only one protocol in the Canary Islands to accompany trans students, in schools.

26. If a person is born with female biological sex, but identifies with the male gender and is sexually attracted to other women, we talk about:

a. A homosexual woman.

b. A straight woman.

c. A homosexual man.

d. A straight man.

27. Should intersex babies be subjected to surgeries?

a. No, never, these procedures are irreversible and result in a body devoid of sexual sensitivity in most cases.

b. Yes, in the case of necessity and once assessed by paediatric surgery.

c. It depends on what the parents decide.
d. It depends on what the doctors and parents think, once the karyotype result arrives.

28. Is it the same being intersex as being trans?

a. Yes, it depends on sexual tastes.
b. Intersexuality is another form of gender diversity.
c. Intersexuality is not a part of gender diversity, because intersexuality is not about gender, nor does it have to do with a transition.
d. Depending on the size of the genitals you are born with; it will be one thing or the other

29. What do you think a cissexual person is?

a. A person who is assigned a sex at birth according to their genitals.
b. A person who is assigned a sex at birth according to their genitals and feels satisfied with that assigned sex.
c. A person who is assigned a sex at birth according to their genitals and does not feel satisfied with that assigned sex.
d. A person who performs a sexual reassignment through surgical procedures.

30. How do intersex and hermaphrodite concepts differ?

a. It is the same, before it was called hermaphrodites and now intersex.
b. The difference lies in the lack of genitals of the intersexual person.
c. The difference is that people with intersex genitals cannot self-fertilize.
d. The difference lies in the size of the genitals, depending on whether it is a smaller size, it will be intersex and a larger size will be considered hermaphroditism.

THANK YOU FOR YOUR COLLABORATION
